# Response to the Letter to the Editor: “Reaffirming Caution—The Unacceptable Vasoconstrictive Risk of CGRP‐Related Therapies in Moyamoya Angiopathy”

**DOI:** 10.1111/ene.70470

**Published:** 2026-01-08

**Authors:** Nicola Rifino, Anne Hege Aamodt, Markus Wiedmann, Markus Kramer, Jana Becker, Stéphanie Guey, Francesco Acerbi, Dominique Herve, Anna Bersano

**Affiliations:** ^1^ Cerebrovascular Unit Fondazione IRCCS Istituto Neurologico Carlo Besta Milan Italy; ^2^ Department of Neurology Oslo University Hospital Oslo Norway; ^3^ Department of Neurosurgery Oslo University Hospital Oslo Norway; ^4^ Department of Neurology Alfried Krupp Hospital Essen Germany; ^5^ CNVT‐CERVCO et département de Neurologie Hôpital Lariboisière, APHP Nord Paris France; ^6^ Department of Neurosurgery Fondazione IRCCS Istituto Neurologico Carlo Besta Milan Italy

Dear Editor,

We thank Drs. Gaul and Naegel for their thoughtful comments on our recent consensus review addressing the spectrum of headaches in Moyamoya angiopathy (MMA). We fully agree that the management of headache in this rare and complex cerebrovascular disorder should be guided by prudence, individualized assessment, and a deep understanding of the disease's unique hemodynamic fragility.

As the authors rightly emphasize, MMA represents a condition in which cerebral perfusion critically depends on compensatory vasodilatory mechanisms that may be highly sensitive to pharmacological modulation [1]. From this perspective, any therapeutic approach acting on vascular tone requires careful consideration, especially when its vascular effects are incompletely understood in such a context.

We would like to clarify that our review did not suggest or encourage the use of calcitonin gene–related peptide (CGRP)–targeted therapies in patients with MMA. Our intention was indeed to acknowledge their relevance in the broader landscape of migraine management, while underscoring that, in the specific setting of MMA, the absence of clinical data and the theoretical implications on cerebrovascular autoregulation call for particular caution.

In fact, as also mentioned by Drs. Gaul and Naegel, the study by Mulder and colleagues [2] provides relevant experimental evidence in this regard. In their murine model of middle cerebral artery occlusion, the authors demonstrated that exposure to small‐molecule CGRP receptor antagonists led to higher rates of cerebral infarction compared with placebo and resulted in larger infarct volumes with longer durations of occlusion. These findings suggest that while CGRP receptor blockade may not independently induce ischemic events, it could potentially exacerbate outcomes when cerebral perfusion is already compromised.

For this reason, pivotal clinical trials of anti‐CGRP therapies systematically excluded patients with cerebrovascular disease, and post‐marketing surveillance has identified occasional cardiovascular adverse events [3, 4].

On the other hand, to thoroughly examine the effect of erenumab on cerebral hemodynamics, one study evaluated cerebral vasomotor reactivity and endothelial function in 60 patients with migraine without aura [5]. The findings revealed no significant alterations following treatment, indicating that in certain contexts, anti‐CGRP antibodies preserve cerebral vasomotor reactivity in migraineurs without aura.

Moreover, current evidence from large randomized controlled trials does not indicate an increased risk of stroke associated with CGRP‐targeted therapies, but data regarding their safety in individuals with preexisting cerebrovascular or cardiovascular disease remain very limited [6].

However, it should be noted that results obtained under physiological conditions may not be generalizable to patients with MMA.

In conclusion, we share the colleagues' view that extreme caution is warranted when considering CGRP‐targeted therapies in patients with MMA. We fully agree that the theoretical risk of impairing compensatory vasodilation cannot be underestimated. However, before formulating strong recommendations that categorically contraindicate these agents, more evidence is needed to define their actual cerebrovascular impact in this specific population.

Until such data become available, as already stated in our review, the use of anti‐CGRP therapies in MMA cannot be recommended, and clinical decisions should remain guided by an individualized, multidisciplinary assessment of risk and benefit.

Sincerely,

Nicola Rifino, MD

on behalf of the co‐authors

## Author Contributions

**Francesco Acerbi:** validation, writing – review and editing. **Anna Bersano:** validation, writing – review and editing. **Anne Hege Aamodt:** writing – review and editing, validation. **Nicola Rifino, Markus Wiedmann, Markus Kramer, Jana Becker, Stephanie Guey, Dominique Herve:** writing – review and editing, validation.

## Conflicts of Interest

The authors declare no conflicts of interest.

## Data Availability

Data sharing not applicable to this article as no datasets were generated or analyzed during the current study.
